# Sustainable Nutritional Strategies for Gut Health in Weaned Pigs: The Role of Reduced Dietary Crude Protein, Organic Acids and Butyrate Production

**DOI:** 10.3390/ani15010066

**Published:** 2024-12-30

**Authors:** Kathryn Ruth Connolly, Torres Sweeney, John V. O’Doherty

**Affiliations:** 1 School of Agriculture and Food Science, University College Dublin, Belfield, D04 W6F6 Dublin, Ireland; ruth.connolly@ucdconnect.ie; 2 School of Veterinary Medicine, University College Dublin, Belfield, D04 W6F6 Dublin, Ireland; torres.sweeney@ucd.ie

**Keywords:** post-weaning, microbiota, gut health, organic acids, grain, crude protein, intestinal dysfunction, antimicrobial resistance, butyrate, swine

## Abstract

Weaning in piglets presents significant physiological and immunological challenges, including gut dysbiosis and increased susceptibility to post-weaning diarrhoea (PWD). Abrupt dietary, environmental, and social changes during this period disrupt the intestinal barrier and microbiota, often necessitating antimicrobial use. Sustainable dietary strategies are critical to addressing these issues while reducing reliance on antimicrobials. Reducing dietary crude protein mitigates the availability of undigested proteins for pathogenic bacteria, lowering harmful by-products like ammonia and branched-chain fatty acids, which exacerbate dysbiosis. Organic acid supplementation improves gastric acidification, nutrient absorption, and microbial balance, while also serving as an energy-efficient alternative to traditional grain preservation methods. Increasing intestinal butyrate, a key short-chain fatty acid with anti-inflammatory and gut-protective properties, is particularly promising. Butyrate strengthens intestinal barrier integrity by upregulating tight junction proteins, reduces inflammation by modulating cytokine responses, and promotes anaerobic microbial stability. Exogenous butyrate supplementation via salts provides immediate benefits, while endogenous stimulation through prebiotics (e.g., resistant starch) and probiotics promotes sustained butyrate production. These interventions selectively enhance butyrate-producing bacteria such as *Roseburia* and *Faecalibacterium prausnitzii*, further stabilising the gut microbiota. Integrating these strategies can enhance gut integrity, microbial resilience, and immune responses in weaned piglets. Their combination offers a sustainable, antimicrobial-free approach to improving health and productivity in modern pig production systems.

## Simple Summary

Weaning is a challenging stage for piglets, involving sudden changes in diet, environment, and social interactions, which place stress on their digestive and immune systems. In commercial pig farming, early weaning often leads to immature gut development, making piglets more vulnerable to health issues like post-weaning diarrhoea. This review discusses natural dietary nutrient strategies to help piglets transition smoothly through weaning without relying on antimicrobials. Lowering crude protein in piglet diets can reduce undigested proteins in the gut, which helps prevent harmful bacteria from causing digestive problems. Adding organic acids to the diet helps maintain a healthy stomach pH, improves digestion, and supports a balanced gut microbiome. Another approach is to boost gut health with butyrate, a beneficial compound that reduces inflammation and protects the gut lining. Butyrate can be provided directly or encouraged naturally by promoting beneficial gut bacteria. These strategies present promising approaches to enhancing piglet health during weaning, although further research is necessary to achieve consistent outcomes. Combining these strategies may provide an effective solution to improving growth, gut health, and the overall sustainability of post-weaned pig production.

## 1. Introduction

Weaning represents a critical developmental phase for piglets, characterised by abrupt dietary, environmental, and social changes that pose significant physiological and immunological challenges. The transition from sow’s milk to solid feed disrupts the gut environment, often leading to impaired digestion, gut dysbiosis, and post-weaning diarrhoea (PWD), a major health and economic challenge in swine production [1,2]. During the suckling phase, piglets rely on lactose as their primary energy source, with its fermentation producing lactic acid that compensates for underdeveloped hydrochloric acid (HCL) secretion. However, the shift to solid feed reduces lactic acid production, leaving gastric pH elevated (approximately 5.0), which is insufficient for optimal pepsin activation and protein digestion [3]. Consequently, undigested proteins reach the distal colon, where they are fermented by nitrogen-utilising bacteria, generating harmful by-products that disrupt gut homeostasis, increase inflammation, and compromise intestinal barrier function [4,5,6].

Traditionally, pharmacological levels of zinc oxide have been widely used in piglet diets to address weaning-related challenges, improving growth performance and reducing the incidence of PWD. However, growing concerns regarding its environmental impact, specifically the accumulation of zinc in soil and water ecosystems, and its role in promoting antimicrobial resistance have led to regulatory bans, including its prohibition in the European Union as of 2022 (Commission Implementing Decision, 2017).

**Figure 1:**
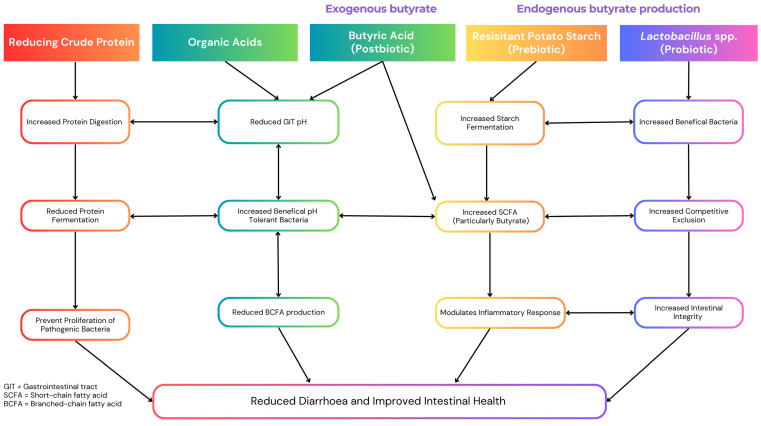
The proposed synergistic effects of reduced dietary crude protein, organic acid and butyrate on post-weaned pig growth and health.

This review evaluates alternative nutritional strategies that address the physiological and microbial challenges associated with weaning, focusing on reducing dietary crude protein (CP), supplementing organic acids, and enhancing intestinal butyrate levels. Reducing CP limits undigested substrates that promote the growth of pathogenic bacteria, while organic acids lower gastric pH, enhance nutrient absorption, and inhibit pathogens. Butyrate, a short-chain fatty acid (SCFA) with potent anti-inflammatory and gut-protective properties, can be supplied directly or promoted endogenously via prebiotics and probiotics, supporting gut integrity and microbial stability. While each of these strategies shows considerable promise when applied individually, their synergistic potential offers a more effective and sustainable solution for mitigating the adverse effects of weaning, as illustrated in Figure 1. This review explores these strategies in detail, proposing a framework for their integration into modern pig production systems to enhance resilience, growth performance, and sustainability.

## 2. Impact of Standard Crude Protein Concentrations in Weaner Pig Diets: Effects on Growth, Gut Health and Immune Function

Weaning is a critical developmental phase in piglets, marked by abrupt dietary changes that exert significant physiological and immunological stress on the gastrointestinal tract. To support rapid growth and maximise feed efficiency during this transition, weaner pig diets are often formulated with high CP levels of 20–23% in pre-starter feeds and 18–20% in starter feeds, incorporating high-quality protein sources such as whey protein, soya bean concentrate, potato protein, and fish meal [7,8]. These diets are designed to meet the piglet’s high amino acid demands, particularly for lysine, while compensating for their limited feed intake [9,10]. However, while high-CP diets are essential for supporting growth, they also present considerable challenges, including heightened risk of PWD, impaired gut health, and suboptimal post-weaning performance [11].

During weaning, stress and reduced feed consumption compromise digestive and immune functions. The immature gut undergoes morphological changes, including villous atrophy, crypt hyperplasia, and reduced activity of brush-border enzymes such as sucrase, lactase, and lipase [12,13]. These changes impair the piglet’s ability to digest and absorb nutrients efficiently. High dietary CP exacerbates this issue by delivering excess undigested protein to the distal intestine, reducing overall digestive efficiency and contributing to gastrointestinal disturbances [14]. In the colon, undigested protein undergoes microbial fermentation, raising colonic pH and promoting the proliferation of pathogenic bacteria such as *Clostridium* and *Bacteroides* [15]. Protein fermentation produces harmful by-products, including ammonia, phenols, and amines, which disrupt gut homeostasis, induce inflammation, and exacerbate PWD [5,16,17]. Additionally, osmotic effects associated with protein fermentation further aggravate diarrhoea by drawing excess water into the intestinal lumen [15]. High-CP diets also alter the gut microbiota by reducing bacterial diversity and enriching pro-inflammatory species such as *Fusobacterium* [18]. These changes increase coliform counts in the colon and faeces while decreasing the beneficial *Lactobacillus*-to-coliform ratio, compounding dysbiosis and intestinal dysfunction [19].

Compounding these microbiological and physiological challenges is the piglets’ underdeveloped immune system, which is further stressed by weaning. Elevated levels of pro-inflammatory cytokines and increased intestinal permeability are common responses to weaning stress [20,21]. High-CP diets can intensify these effects by promoting allergic reactions and inflammatory responses, further exacerbating gastrointestinal disturbances and PWD [22]. Effective protein digestion, facilitated by sufficient digestive enzyme activity and bile salt production, is essential for breaking down dietary proteins into non-immunogenic fragments and mitigating these effects [23].

### The Effects of Low Crude Protein in Weaner Pig Diets: Diarrhoea, Gut Health, Immune Function and Growth

Managing CP levels plays a pivotal role in enhancing intestinal health and reducing the incidence of PWD in piglets. Extensive research has demonstrated that lowering CP levels in piglet diets effectively limits the amount of undigested protein reaching the gastrointestinal tract (GIT). This reduces protein fermentation, thereby minimising the production of harmful by-products such as ammonia, amines, and polyamines [19,24,25,26], which damage colonic epithelial cells and decrease villous height in the small intestine, significantly impairing nutrient absorption [16,27]. Furthermore, elevated ammonia levels disrupt the oxidative metabolism of SCFAs, reducing energy availability in the large intestine and compromising its overall functionality [5]. Hence, lowering dietary CP levels is an important strategy for improving gut health and optimising post-weaning performance in piglets.

Numerous studies have highlighted the benefits of low-CP diets supplemented with amino acids in weaned piglets, which have been highlighted in Figure 1. For example, reduced diarrhoea scores and increased SCFA production was observed in piglets fed a 16% CP diet supplemented with amino acids and probiotics compared to a 20% CP diet [28]. Similarly, a 14% CP diet with essential amino acid supplementation enhanced pancreatic enzyme expression compared to a 17% CP diet and a 20% CP diet [29], while reduced ammonia concentrations and shorter crypt depths were reported in pigs fed a 19% CP diet compared to those offered a 21% CP diet [30]. However, reducing CP levels to 17% or lower may impair growth performance by decreasing villous height and reducing pancreatic function, which can compromise nutrient absorption [31,32].

Additionally, low-CP diets have been shown to promote beneficial microbial populations, such as butyrate-producing bacteria like *Roseburia* and *Eubacterium rectale*, which support gut health and reduce inflammation [5]. Piglets on a 15.5% CP diet had higher abundances of anti-inflammatory bacteria (*Succinivibrionaceae*), fibre-degrading bacteria (*Fibrobacteraceae*), and *Lactobacillus*, indicating a more stable and beneficial microbiome [33]. However, excessively low CP levels lower SCFA butyrate production, potentially compromising gut health [34]. These results suggest that microbial responses to CP levels can vary depending on factors such as sanitation, genetics, social stress, or the protein source used in diets.

Low-CP diets also have implications for immune modulation. High-CP diets have been shown to suppress the expression of the MCT1 gene, which is crucial for mucus production and gut barrier function. Conversely, low-CP diets stimulate MCT1 expression, which is enhanced by butyrate and helps control intestinal inflammation [5]. Piglets on a low-CP diet exhibited reduced expression of pro-inflammatory cytokines in the colon, correlating with lower levels of ammonia and *E. coli* [35]. Additionally, moderate reductions in CP levels have been reported to increase the expression of tight junction proteins, strengthening gut integrity [36]. However, certain low-CP diets have been associated with increased inflammation, particularly in the presence of soya antigens or synthetic amino acids. Elevated pro-inflammatory markers were observed in piglets on an 18% low-CP diet, likely due to heightened sensitivity to dietary antigens [37]. Furthermore, synthetic amino acids may lack the ability to bind to immune cells, unlike natural proteins, potentially failing to stimulate regulatory immune responses and exacerbating inflammation [38]. Variability in feed ingredient quality, feeding patterns, and environmental conditions complicates the consistent implementation of low-CP diets [27].

A significant challenge of low-CP diets is maintaining growth performance. When CP levels are reduced, careful dietary formulation is required to ensure amino acid requirements are met [27]. It is recommended to supplement with the first four limiting amino acids or branched-chain amino acids when CP levels are lowered below 3% of the NRC requirements, and with dietary nitrogen or non-essential amino acids (NEAAs) when CP levels are lowered below 6% of the NRC requirements [27]. Lowering CP levels can limit the availability of nitrogen for synthesising NEAAs, which are essential for protein synthesis and growth. However, variations in feed ingredients, feeding patterns, and environmental conditions can make it difficult to achieve consistent results [27]. This variability is a major limitation in the widespread adoption of low-CP diets. Supporting this, studies report mixed results. For example, reduced growth performance has been reported when CP levels were lowered to 17% and 12.7% [31,39]. Conversely, reducing CP levels to 18% maintained growth performance, with further reductions improving feed efficiency [14,40].

Balancing CP levels in piglet diets remains critical for optimising growth, gut health, and immune function while minimising PWD. Although reducing CP levels promotes microbiome stability and reduces inflammation, care must be taken to avoid compromising growth and digestive performance. Fine-tuning CP levels with precise amino acid supplementation is crucial for developing optimal nutritional strategies for weaned piglets. Reducing CP levels offers benefits such as reduced nitrogen excretion, improved gut health, and decreased risk of PWD. However, achieving these benefits while maintaining growth performance remains challenging due to the need for balanced nitrogen and amino acid availability. The effects of lowering dietary CP on pig growth performance, diarrhoea scores, and intestinal health and function are summarised in Table 1.

## 3. The Role of Organic Acids in Weaner Pig Diets

The inclusion of organic acids in weaning piglet diets has proven to be an effective strategy for improving growth performance, stimulating feed intake, enhancing gut health, and bolstering resilience during the critical post-weaning period, with their mechanisms outlined in Figure 1. Organic acids can be classified into three primary categories: SCFAs, medium-chain fatty acids (MCFAs), and tricarboxylic acids (TCAs). These acids are added to diets either individually or as blends, in free forms or as salts [41]. As naturally occurring cellular metabolites, organic acids have low toxicity and are well tolerated by animals [42]. Short-chain fatty acids, such as acetic, propionic, and butyric acids (≤5 carbons), are particularly important for maintaining intestinal morphology and barrier integrity [43]. Medium-chain fatty acids (7–12 carbons) act as efficient energy sources [44], while TCAs, intermediates of the Krebs cycle, positively influence gut barrier function, intestinal microbiome balance, and structural integrity [43].

Piglets exhibit a natural preference for sour tastes, making organic acids like tartaric and citric acids appealing dietary supplements [42]. The addition of 1.6% lactic acid to piglet diets significantly increases average daily feed intake (ADFI), comparable to diets containing antibiotics such as lincospectin (lincomycin and spectinomycin) [45]. Organic acid supplementation generally enhances ADFI compared to non-supplemented diets, although excessive concentrations of acids like acetic, propionic, or formic acids can cause discomfort, reducing feed intake [46]. This highlights that the efficacy of organic acids depends on factors such as acid type, inclusion levels, and interactions with other dietary components.

One of the major physiological challenges during weaning is the increase in gastric pH, which compromises the stomach’s role as a pathogen barrier [47]. Before weaning, bacteria such as *Streptococcus suis* constitute less than 4% of the stomach’s microbiome, while *Lactobacillus* represents approximately 14% [48]. Post-weaning, the proportion of *S. suis* increases, while *Lactobacillus* levels decrease, increasing the pathogenic load throughout the GIT. High gastric pH not only facilitates bacterial overgrowth but also impairs protein digestion and the absorption of nutrients like calcium, iron, and vitamin B12 [49]. Organic acids, with their low acid-binding capacities, help reduce dietary and gastric pH, improving digestion and suppressing pathogen growth in post-weaned piglets [50]. However, research on the effects of organic acids on gastric pH shows mixed results. While lactic and formic acids have been shown to reduce stomach pH in weaned pigs [51], other studies found no significant impact of certain organic acids or blends on gastric pH [52]. These variations suggest that the benefits of organic acids extend beyond pH reduction and include mechanisms such as improved nutrient digestibility. For instance, organic acid supplementation has been shown to enhance pancreatic enzyme production and increase the digestibility of amino acids like methionine, lysine, and leucine [53]. By lowering pH, organic acids can also shorten feed transit time, allowing for more efficient nutrient absorption and better utilisation of minerals like phosphorus, zinc, and calcium [3,54].

The effects of organic acids on intestinal morphology are similarly variable. Some studies report no changes in gut structure with organic acid supplementation [55,56], while others demonstrate improvements in villous height-to-crypt depth ratios, indicating enhanced nutrient absorption [57,58].

Beyond their digestive benefits, organic acids also modulate the microbiome. They create an acidic environment that is unfavourable for pathogens like *E. coli* and *Clostridia* while supporting beneficial bacteria such as *Lactobacillus* and SCFA-producing bacteria [3,59]. For example, piglets supplemented with organic acids, MCFAs, and phenolic compounds exhibited an increased abundance of beneficial bacterial families, including *Lachnospiraceae* and *Lactobacillaceae* [60]. These bacteria are associated with anti-inflammatory effects, improved immune function, and tight junction integrity [61]. However, the efficacy of organic acids as microbiome modulators varies with their chain length, saturation, and inclusion levels [41].

Despite their benefits, the inappropriate use of organic acids can lead to unintended effects. For instance, high levels of potassium diformate (1.8%) can reduce beneficial lactic acid bacteria in faeces, emphasising the need for precise formulation [62]. Overall, incorporating organic acids into piglet diets offers significant potential for improving gut health, nutrient utilisation, and microbiome balance, but their application must be carefully tailored to optimise benefits while minimising drawbacks. The effects of organic acid supplementation on pig growth performance, diarrhoea scores, and intestinal health and function are presented in Table 2.

### The Potential of Organic Acid-Preserved Grain in Weaner Diets: Enhancing Grain Quality, Gut Health, Growth and Sustainability

Grain preservation is a fundamental aspect of animal feed production, as it ensures the maintenance of feed quality while preventing spoilage. Traditional grain drying methods, though widely adopted, are unsustainably energy-intensive and often lead to uneven moisture distribution, which fosters mould growth and increases the risk of mycotoxin contamination [64,65]. This is particularly detrimental in piglet nutrition, as mycotoxins are known to impair growth, compromise immune function, and negatively affect overall health, thereby reducing productivity [66].

Organic acids have emerged as a versatile solution in feed preservation, offering a more energy-efficient alternative to traditional drying processes. Preserving grain using organic acid is an uncomplicated process. Initially, the grain is emptied into a screw conveyor, where the acid is applied at the desired concentration. The grain and acid are then mixed thoroughly before storage in a corrosive-resistant storage facility. By lowering grain pH and inhibiting fungal proliferation, organic acids such as propionic acid effectively prevent mould growth and mycotoxin formation, preserving feed quality and extending shelf life [67]. Moreover, organic acid-preserved grains have demonstrated superior nutritional and performance benefits compared to heat-dried grains. Supplementing organic acid-preserved grains exhibited higher digestible and metabolisable energy values, contributing to improved average daily gain (ADG) and ADFI in pigs [68]. Similarly, piglets fed organic acid-preserved grain exhibited greater growth performance compared to those receiving the same organic acid blend as a dietary additive [69], emphasising the added nutritional value of preserved grains.

Beyond nutritional benefits, organic acid-preserved grains positively impact piglet health. Weaned pigs consuming organic acid-preserved grain had enhanced growth performance, improved nutrient digestibility, and increased proliferation of beneficial gut microbiota, such as *Faecalibacterium* [70]. These findings suggest that organic acid preservation not only improves feed quality but also enhances post-weaning gastrointestinal health and resilience.

The adoption of organic acid-preserved grains has the potential to promote sustainable swine production by reducing microbial contamination in feed, improving nutritional value, and directly supporting gut health through exclusion of pathogen microbial populations and nutrient optimisation. However, further research is warranted to evaluate the long-term effects of organic acid-preserved grains on intestinal health, growth performance, and interactions with other dietary strategies. Such investigations will provide a deeper understanding of their role in enhancing piglet performance and advancing sustainable livestock production practices.

## 4. The Protective Effects of Butyrate

Gut dysbiosis during the weaning phase disrupts the proliferation of butyrate-producing bacteria, significantly impairing the intestinal health and resilience of weaned piglets. This imbalance is characterised by an overgrowth of facultative anaerobic pathogens such as *E. coli* and *Salmonella* and a corresponding decline in beneficial butyrate-producing bacteria like *Roseburia* and *Faecalibacterium prausnitzii* [56,71,72,73]. Butyrate, a key SCFA, plays a critical role in maintaining intestinal homeostasis, promoting gut barrier integrity, regulating immune responses, and providing energy to colonocytes. Enhancing butyrate levels in weaned pigs through dietary strategies is a promising solution to mitigate post-weaning challenges such as PWD, inflammation, and compromised growth performance. Butyrate provides up to 70% of the energy requirements of colonocytes, making it vital for maintaining the structural integrity and regenerative capacity of the intestinal lining [74,75,76]. Colonocytes absorb butyrate through transporters such as SLC16A1 and SLC5A8, which facilitate its rapid oxidation to generate ATP. This energy supports the maintenance of tight junctions, crucial for preventing pathogen translocation and maintaining intestinal barrier function. In addition to its role as an energy source, butyrate has profound immunomodulatory effects. It suppresses intestinal tissue pro-inflammatory cytokines such as IL-6, CXCL8,TNF-α, and IFN-γ, while upregulating anti-inflammatory markers like IL-10 and TGF-β [77]. These actions reduce intestinal inflammation, support immune tolerance, and promote resilience against weaning-associated stress. Butyrate also preserves an anaerobic gut environment, which is critical for maintaining a healthy microbiota. Anaerobic bacteria dominate the microbiota under homeostatic conditions, effectively suppressing facultative anaerobes, including pathogens like *E. coli* and *Salmonella*. However, during dysbiosis, facultative anaerobes proliferate, further disrupting the microbial balance and outcompeting butyrate producers [72]. By fostering an anaerobic environment, butyrate indirectly supports beneficial microbes and strengthens intestinal defences against infections.

### 4.1. The Effects of Supplementing Exogenous Butyrate on Post-Weaned Pig Growth and Gut Health

Strategies to enhance butyrate levels include exogenous supplementation with butyrate salts and promoting endogenous butyrate production through microbial fermentation. Exogenous supplementation typically uses sodium, potassium, magnesium, or calcium butyrate, which are less odorous and more practical for animal feed than pure butyric acid [78]. Exogenous butyrate supplementation has exhibited varying impacts on the performance of weaned piglets, with its proposed mechanism of action detailed in Figure 1. Sodium butyrate at 0.8 g/kg was reported to increased ADG by 20% and feed intake by 16% during the first 15 days post-weaning [79]. Similarly, 2000 mg/kg sodium butyrate improved gut barrier integrity by increasing the expression of tight junction proteins such as occludin, claudin-3, and ZO-1, reducing intestinal permeability and diarrhoea [80]. However, not all studies report consistent benefits. Sodium butyrate provided no significant effects on growth performance or mucosal morphology, although it increased caecal pH and ammonia concentrations [81]. Coated sodium butyrate at 500–1000 ppm was found to reduce feed intake, emphasising the need for optimising dosages and delivery method [82].

### 4.2. Promoting Endogenous Butyrate Production in Weaned Pigs via Prebiotic and Probiotic Supplementation: Effects on Growth, Gut Health and the Gut Microbiome

Endogenous butyrate production relies on the fermentation of dietary fibres and prebiotics, with resistant starch (RS) being particularly effective. Resistant starch, especially RS2 (e.g., raw potato starch), resists enzymatic digestion in the small intestine and reaches the colon intact, where it undergoes fermentation by butyrate-producing bacteria such as *Roseburia* and *Faecalibacterium prausnitzii* [83]. This fermentation process promotes butyrate synthesis while reducing harmful by-products like branched-chain fatty acids and ammonia, which are associated with proteolytic fermentation [17,84]. Studies demonstrate that low to moderate RS supplementation (0.5–5%) optimises butyrate production, supports beneficial microbial populations, and improves overall gut health [85,86]. Additionally, RS enhances gut barrier function by increasing the expression of MUC2, a critical mucin protein, and IgA secretion, strengthening mucosal defences [87]. Excessive RS supplementation (≥7%), however, can disrupt microbial balance, reduce butyrate synthesis, and exacerbate diarrhoea [88,89]. This emphasises the importance of precise inclusion to achieve optimal benefits without adverse effects.

Probiotics enhance the effectiveness of RS in promoting butyrate production by creating a gut environment conducive to fermentation, as illustrated in Figure 1. Strains such as *Lactobacillus plantarum* and *Lactobacillus reuteri* produce lactate and acetate, which act as precursors for butyrate synthesis via cross-feeding with butyrate-producing bacteria [90]. For instance, supplementation with *Lactobacillus plantarum* has been shown to increase colonic butyrate concentrations and promote the growth of beneficial species like *Roseburia* spp. and *Faecalibacterium prausnitzii* [91]. Probiotics also strengthen gut barrier integrity by upregulating tight junction proteins, reducing intestinal permeability, and alleviating damage caused by weaning stress [92,93]. Their immunomodulatory effects, including increased IL-10 and TGF-β1 production, further enhance the gut environment for sustained butyrate synthesis [94].

The combination of RS and probiotics presents a synergistic strategy for improving gut health. RS provides fermentable substrates for butyrate production, while probiotics stabilise the gut microbiota and facilitate cross-feeding interactions. For example, co-supplementation with RS and *Lactobacillus plantarum* has been shown to elevate SCFA levels, fortify the gut barrier, and reduce intestinal inflammation during weaning stress [90]. However, balancing RS and probiotics is crucial, as excessive supplementation can disrupt microbial communities and reduce butyrate synthesis [88,95].

Boosting butyrate production through RS and probiotics offers a sustainable, long-lasting solution for improving gut health, immune function, and growth performance in weaned piglets. This approach effectively addresses challenges such as PWD and intestinal inflammation while supporting piglet development and overall resilience.

## 5. Exploring the Synergistic Effects of Combining These Dietary Strategies for Optimal Health and Growth in Weaner Pigs

A review of the literature indicates that strategies such as reducing dietary CP levels, incorporating organic acids, and enhancing intestinal butyrate levels—either through exogenous supplementation or by promoting endogenous production with pre- and probiotics—are promising approaches to mitigate the physiological challenges associated with weaning in piglets. These strategies show significant potential for improving growth performance and intestinal health without relying on antimicrobials. However, the inconsistencies and variabilities in their outcomes present challenges for consistent application in pig production systems. Despite the complementary effects of these strategies, there remains a notable gap in the literature exploring their combined implementation, which could reduce individual limitations and enhance overall efficacy.

For instance, the use of organic acid-preserved grain may provide additional benefits compared to traditional organic acid supplementation by improving grain quality while directly influencing gastrointestinal health and function. Furthermore, integrating organic acid-preserved grain into low-CP diets could mitigate the potential negative effects of reduced CP levels on growth performance by improving protein digestive efficiency, as demonstrated in broiler studies [96]. Similarly, incorporating butyrate supplementation into low-CP diets could bolster intestinal fermentative capacity and compensate for the reduced butyrate production often observed in pigs consuming low-CP diets [34]. A combination of exogenous butyrate supplementation with endogenous butyrate-promoting additives, such as RS and *Lactobacillus* spp., presents an innovative opportunity. This approach could not only enhance initial intestinal butyrate levels post-weaning to counteract the negative effects of weaning, but also prime the intestine for sustained, lifelong butyrate production.

The interconnected mechanisms of these dietary strategies suggest that their combined application could improve post-weaning growth performance and intestinal health while addressing the limitations associated with each individual approach. The potential synergistic effects of these strategies, as depicted in Figure 1, highlight the importance of further research to identify the most effective combinations for optimising gut health and growth performance in weaned piglets. Identifying the optimal integration of these approaches could significantly enhance post-weaning pig production, providing a robust and sustainable alternative to conventional antimicrobial use.

## 6. Conclusions

In conclusion, lowering dietary CP levels effectively reduces the availability of undigested substrates in the gut, limiting the proliferation of pathogenic bacteria and mitigating PWD. This approach improves gastrointestinal health and promotes a more stable gut microbiome. The incorporation of organic acids further supports gut health by lowering gastric pH, inhibiting harmful bacteria, enhancing nutrient digestibility, and fostering the growth of beneficial microbial populations. Strategies to increase gut butyrate levels, whether through direct supplementation or by stimulating endogenous production, provide critical benefits, including anti-inflammatory effects, improved intestinal integrity, and enhanced immune function, which collectively strengthen the piglet’s resilience during the post-weaning period. Integrating these approaches offers a sustainable and multifaceted strategy to improve weaning outcomes by optimising nutrient utilisation, enhancing growth performance, and promoting a balanced intestinal environment while reducing reliance on antimicrobials. These interventions have the potential to address the physiological and microbial challenges of weaning and support lifelong gut health.

However, the effectiveness of these strategies can be influenced by various factors, including dietary composition, production environments, and individual piglet variability. Further research is needed to refine their application, optimise dosage and timing, and explore interactions between strategies to maximise their synergistic potential. Advancing these nutritional approaches is essential for improving piglet health and welfare, supporting productivity, and promoting sustainability in swine production systems, ultimately contributing to a more resilient and innovative livestock industry.

## Figures and Tables

**Table 1:** The effect of dietary crude protein levels on growth performance, diarrhoea scores, and intestinal health and function of pigs.

Pig Age	Control CP Level	Low CP Level	Synthetic Amino Acid Supplementation	Growth Performance and Diarrhoea	Intestinal Health and Function	Ref
28 days	23% CP	18% CP 13% CP	Lysine, methionine, threonine, tryptophan	Reducing CP reduced ADG and G:F Reducing CP reduced FS	Reducing CP decreased coliform numbers in the proximal colon and faeces and increased lactobacillus: coliform ratio in proximal colon	[19]
21 days	25.6% CP for 14 days	17.5% CP for 7 days 17.5% CP for 14 days with 107 cfu/*E. coli* 17.5% CP for 14 days without 107 cfu/*E. coli*	Isoleucine and valine	No effect of low-CP diets on ADG, ADFI or G:F	Low-CP diets decreased faecal ammonia and plasma nitrogen concentrations Low-CP diets reduced diarrhoea and increased faecal DM Low-CP diets reduced total faecal VFA concentrations	[24]
21 days	23% CP (HP) 23% CP with 2.5 g/kg lincospectin and 3.0 g/kg zinc oxide (HP + AMC)	18.5% CP without essential amino acid supplementation (RP) 18.5% CP with amino acid supplementation (RP + AA)	Lysine, methionine, threonine, isoleucine, tryptophan and valine	RP + AA diet reduced FS compared to HP + AMC diet. RP diet reduced growth and F:G RP + AA diet grew comparably to HP Diet and HP + AMC diet	On day 8 post-weaning, RP + AA had reduced PUN levels compared to other diets	[25]
18 days	23.1% CP	21.2% CP 18.9% CP 17.2% CP	Lysine, threonine, tryptophan, methionine, isoleucine, valine, histidine and phenylalanine	Reducing CP caused a linear decrease in ADG and a quadratic decrease in G:F ratio Reducing CP linearly improved faecal consistency	17.2% CP reduced duodenal and jejunal VH compared to the 23.1% CP Sucrase and lactase activities reduced in proximal jejunum as CP levels decreased	[26]
35 days	20% CP	17% CP 14% CP	Lysine, methionine, threonine and tryptophan	14% CP diet had reduced ADFI compared to 17% and 20% CP diets Reducing CP levels reduced ADG F:G was lower in the 17% and 14% CP diets compared to the 20% CP	The expression of jejunal amylase was highest in 20% CP diet The expression of jejunal maltase was highest in 17% CP diet The expression of GPR93 was higher in the small intestine 14% CP and 17% CP diets than the 20% CP diet The expression of pancreatic lipase and elastase were higher in pigs offered low-CP diets than the 20% CP diet	[29]
17 days	21% CP	19% CP 19% CP deficient in isoleucine 17% supplemented with amino acids	17% CP diet supplemented with isoleucine and valine	The low-CP diets had reduced ADG and F:G compared to the 21% CP diet The 17% CP diet had the highest ADFI compared to all other treatments The low-CP diets had lower FS compared to the 21% CP diet	The 17% CP diet had lower small intestine weight compared to the 21% CP diet The 21% CP diet had deeper ileal crypts compared to all other treatments The low-CP diets had reduced caecal luminal ammonia N concentrations compared to other diets	[30]
21 days	20.7% CP	16.7% CP 12.7% CP	Lysine, methionine, threonine, tryptophan, leucine, isoleucine and valine	The 16.7% CP and 12.7% CP diets had lower ADG and F:G than the 20.7% CP diet No effect of CP level on ADFI	The relative weights of pancreas (g/kg BW) decreased as CP levels decreased Reducing CP levels reduced protein synthesis in the pancreas, liver, kidney and longissimus muscle	[31]
Weaned pigs (9.57 ± 0.61 kg) BW	20% CP (NP)	17% CP (MP) 14% CP (LP)	Lysine, methionine, threonine and tryptophan	Final BW of LP diet was lower than NP diet LP group had lower ADFI and ADG compared to NP diet The F:G was higher in LP diet compared to NP diet	Duodenal and jejunal VH reduced as CP levels reduced Pepsin activity in the stomach decreased as CP levels decreased Blood urea nitrogen was lower as CP levels reduced Reducing CP levels had no effect on growth hormones, insulin, jejunal or colonic microbiome and VFA concentrations	[32]
25 days	17.5% CP (HP)	15.5% CP (LP)	Lysine, methionine, valine, tryptophan and threonine	LP diet had reduced ADG and F:G LP diet had lower FS	LP diet had higher relative abundance of Fibrobacteres, Proteobacteria and Spirochaetes in faeces	[33]
28 days	21% CP 21% CP +ZnO (3100 ppm reduced to 1500 ppm after 14 days 21% CP + 300 ppm laminarin	18% CP 18% CP+ ZnO (3100 ppm reduced to 1500 ppm after 14 days) 18% CP + 300 ppm laminarin	Lysine, methionine, threonine, tryptophan and valine	**Sanitary Conditions:** 18% CP increased the incidence of diarrhoea CP No effect of 18% CP on growth performance **Unsanitary Conditions:** No effect of 18% CP on growth performance 18% CP reduced FS	**Sanitary Conditions:** 18% CP reduced duodenal *AMY2*, *SLC2A7* and increased *SLC16A1* 18% CP reduced jejunal *SLC2A7* and *SLC16A1* 18% CP increased ileal *SLC16A1* + reduced *SLC2A2*, *SLC5A1* and *SLC6A19* 18% CP increased jejunal *IL1A*, *IL1B*, *CXCL8* and *TLR4* 18% CP increased ileal *IL6*, *CXCL8*, *NFKB1*, *IL1A*, *IL1B*, *TGFB1*, *TNF*, *TLR2* and *TLR4* 18% CP increased colonic *Enterobacteriaceae* **Unsanitary Conditions:** No effect of 18% CP on faecal microbiome	[37]
42 days	20% CP from days 42–77 18% CP from days 77–120	16% CP from days 42–77 14% CP from days 77–120	Lysine, methionine, threonine and tryptophan	No effect of CP on growth performance throughout entire experiment	**Day 77** Low CP increased colonic propionate and butyrate and reduced ammonia and phenol concentrations Low CP increased colonic *Clostridium cluster IV* and *Clostridium cluster XIVa* counts and reduced *E. coli* counts Low CP decreased mRNA levels of *TLR4*, *IFN*-γ and *TNF*-α in the colonic mucosa	[35]

ADG = average daily fain, ADFI = average daily feed intake, BW = body weight, CP = crude protein, DM = dry matter, G:F = gain to feed ratio, FS = faecal scores, PUN = plasma urea nitrogen, VFA = volatile fatty acids, VH = villous height.

**Table 2:** The effect of organic acid supplementation on the growth performance, diarrhoea scores, and intestinal health and function of pigs.

Pig Age	Organic Acid and Inclusion Level	Growth Performance and Diarrhoea	Intestinal Health and Function	Ref
25 days	Propionic acid (1%) Lactic acid (1.6%) Formic acid (1.2%) Malic acid (1.2%) Citric acid (1.5%) Fumaric acid (1.5%)	All OA treatments had reduced incidence and severity of diarrhoea All OA treatments had heavier final BW All OA treatments had higher ADFI Lactic acid treatment had higher ADG compared to other OA treatments and negative control	Faecal ETEC were undetectable in OA treatments	[45]
21 days	Sodium diformate (1.2%)	Day 0–7, sodium diformate increased ADG and d 7 BW Sodium diformate improved ADF and G:F from d 7–21 and d 0–21 No main effect of sodium diformate on faecal DM	No markers of intestinal health and function collected	[47]
Weaned pigs (8.4 ± 0.8 kg) BW	Potassium diformate (0.5%) Organic acid blend (ASD) (0.5%) **Oral *E. coli* K88 challenge** Organic acid blend (ASD) (0.5% and 1%)	ASD improved ADG and F:G **Oral *E. coli* K88 challenge** Day 5–14 post-challenge, 0.5% and 1% ASD improved ADG and F:G	Pigs offered potassium diformate and ASD had increased faecal *Lactobacilli* counts **Oral *E. coli* K88 challenge** No effect of ASD on digesta pH 0.5% ASD increased duodenal lactobacilli compared to control	[63]
**Experiment 1** 21 days **Experiment 2** 28 days	**Experiment 1 + 2** OA and MCFA blend (calcium formate, calcium lactate, citric acid and MCFA) (34%, 16%, 70% and 13% respectively)	**Experiment 1** OA and MCFA blend increased ADFI in first two weeks OA and MCFA blend increased BW at end of 4-week experimental period and improved F:G	**Experiment 2** OA and MCFA blend had higher apparent ileal digestibility of methionine, lysine, threonine, valine, phenylalanine, leucine, isoleucine, histidine, aspartic acid, glutamic acid, serine and tyrosine Jejunal mRNA abundance of AA transporters *EAAT3* and *CAT2* were higher in OA and MCFA blend group OA and MCFA blend had higher ileal and rectal *Lactobacillus* and higher rectal total bacteria counts	[53]
25 days	Short-chain OA (0.41% fumaric acid and 0.32% lactic acid) MCFA (0.15% caprylic and capric acid) Combination of short-chain OA and MCFA	Not recorded	No effect of short-chain OA or MCFA on jejunal morphology The short-chain OA increased CD^2−^ and CD^8−^ in jejunal epithelium	[55]
**Experiment 1 + 2** 21 days	**Experiment 1** OA1 (synergistic blend of phenolic compounds, slow release C12, target release butyrate, MCFAs and free and buffered OA) (0.2%) OA2 (blend of free and buffered short-chain fatty acids combined with MCFAs) (0.3%) Combination of OA1 (0.2%) and OA2 (0.3%) **Experiment 2** OA1 (0.2%) OA3 (synergistic blend of free and buffered OA based on formic acid) (0.3%) Combination of OA1 (02%) and OA3 (0.3%)	**Experiment 1** No effect of OA1 or OA2 on growth performance OA1 and OA2 reduced diarrhoea index from d 15–17 **Experiment 2** OA1, OA3 and combination of OA1 and OA3 had improved ADG and F: G OA1, OA3 and combination of OA1 and OA3 reduced diarrhoea index	**Experiment 1** No effect of OA1 or OA2 on pH in stomach, jejunal or colonic digesta OA2 increased duodenal VH Combination of OA1 and OA2 increased acetate and propionic concentrations in caecum and colon compared to control OA2 reduced *E. coli* numbers in colon **Experiment 2** No effect of OA1 or OA3 on pH in stomach, duodenal, jejunal, ileal of colonic digesta OA1 and OA3 increased ileal VH OA1 and OA3 combination increased colonic acetic and propionic concentrations OA1 and OA3 combination increased colonic *Prevotella*	[56]
Weaned pigs (8.6 ± 1.56 kg)	OA1 (synergistic blend of free and buffered short chain fatty acids combined with MCFA) (3000 mg/kg) OA2 (synergistic blend of phenolic compound, slow release C12, target release butyrate and sorbic acid, MCFA and OA) (2000 mg/kg)	OA2 increased ADG, and F:G for overall experimental period OA1 improved F:G for overall experimental period Both OAs reduced diarrhoea rate in phase 1 (day 1–14) and overall experimental period	OA1 and OA2 increased faecal SCFA concentrations OA1 and OA2 increased acetic, propionic and isobutyric content in faeces OA2 increased apparent total tract digestibility of NDF, ADF and phosphorus in phase 1 OA1 increased apparent total tract digestibility of DM, NDF and ADF in phase 2 (day 14–28) OA1 increased serum IgM OA1 and OA2 reduced serum H2O2 in phase 1 OA2 reduced jejunal CD	[57]
21 days	3 g/kg sodium butyrate 3 g/kg lauric acid 3 g/kg stearic acid	No effect of ADG, ADFI or G:F for day 1–14 or 1–28 Sodium butyrate increased G:F from days 15–28	Stearic acid and sodium butyrate increased ileal VH compared to control Sodium butyrate increased jejunal CD compared to control	[58]
28 days	Potassium diformate 1.8%	No effect of potassium diformate on growth performance or diarrhoea	No effect of potassium diformate on gastrointestinal pH Potassium difomate reduced faecal concentrations of lactic acid bacteria on d 5, 14, 21 and 28	[62]

ADG = average daily gain, ADFI = average daily feed intake, BW = body weight, CD = crypt depth, d = day, DM = dry matter, G:F = gain to feed ratio, OA = organic acids, SCFA = short chain fatty acids, VH = villous height.

## Data Availability

No new data were created or analysed in this study.
